# Combined Machine Learning and Semantic Modelling for Situation Awareness and Healthcare Decision Support

**DOI:** 10.1007/978-3-030-51517-1_16

**Published:** 2020-05-31

**Authors:** Amira Henaien, Hadda Ben Elhadj, Lamia Chaari Fourati

**Affiliations:** 8 Digital Research Centre of Sfax, Sfax, Tunisia; 9 Institut Mines-Télécom, CNRS, Paris, France; 10 Université de Sherbrooke, Sherbrooke, QC Canada; 11 Digital Research Centre of Sfax, Sfax, Tunisia; 12 University of Sfax, Sfax, Tunisia; 13 King Khalid University, Abha, Kingdom of Saudi Arabia; 14 Laboratory of Technology and Smart Systems (LT2S), LR16CRNS01, Digital Research Center of Sfax, Sfax, Tunisia

**Keywords:** Active and assisted living, Ontologies, ML, Health monitoring, Preventive personalized health services

## Abstract

The average of global life expectancy at birth was 72 years in 2016 [1], however, the global *healthy* life expectancy at birth was only 63.3 years in the same year, 2016 [2]. Living a long life is not any more as challenging as assuring active and associated life [25]. We propose in this paper an IoT based holistic remote health monitoring system for chronically ill and elderly patients. It supports smart clinical decision help and prediction. The patient heterogeneous vital signs and contexts gathered from wore and surrounding sensors are semantically simplified and modeled via a validated ontology composed by FOAF (Friend of a Friend), SSN (Semantic Sensors Network)/SOSA (Sensor, Observation, Sample and Actuator) and ICNP (International Classification Nursing Practices) ontologies. The reasoner engine is based on a scalable set of inference rules cohesively integrated with a ML (Machine Learning) algorithm to ensure predictive analytic and preventive personalized health services. Experimental results prove the efficiency of the proposed system.

## Introduction

Information revolution and wireless mobile technology growth have made a considerable contribution to the expansion and empowering of E-health services. In fact, smart remote and mobile healthcare applications are making an enormous shift in the health and social care workforce efficiency as well as patients’ well-being. The main target of such applications is leveraging IoT, ML and Semantic Web technologies to ensure opportunities that enable people to be and do what they value throughout their lives despite illness. The headline goal of E-health is promoting elderly independence and sustaining cognitive and physical capability via multidisciplinary and user-friendly technology. [6] is one of the earliest studies that has highlighted the importance of the development of a powerful healthcare system. The study conclude that an integrated multidisciplinary infrastructure allowing interoperability and scalability is crucial. From that stage to nowadays, innovations in Information and Communication Technologies have radically changed healthcare services, created several manners for collecting and managing data effectively and provided several solutions to e-healthcare challenges [11]. The health sector is nowadays in its knowledge age: data, information, and knowledge are used in real time to support effective integration of prevention, treatment, and recovery services across healthcare services. Computers are not only used to provide health services but also to improve health itself through the management of the knowledge base and the automatic support of decisions. Therefore, healthcare applications are now exchanging and performing not only an enormous volume of data but also an important quantity of information and a large knowledge base Fig. 2. Thus, the semantic interoperability is becoming a crucial feature that is hard to imagine a healthcare or clinical system architecture without it [15]. The IoT ontologies appear as a suitable alternative to exchange knowledge per providing the required semantics to augment the data contained in the information model, and that to support service management operations [32]. Ontological models are becoming commonly used models in healthcare systems providing a flexible approach to integrate data and share meaning and able to assist inferring meaning [24, 33]. Often ontology-based systems are using rule-based decision support system in order to assure an active and assisted monitors of patients [33]. However, a majority of those systems are not performing an automatic updates of the knowledge base. Hence, we propose in this paper a semantic-based healthcare monitoring system with seamless integration of many intricate existing knowledge, ontologies and ML technologies. It is a dynamic rule-based system, which infers information and medical recommendations based on the interaction of IoT input captured data, subjective and objective knowledge and a dynamic rule base updates by a ML algorithm based rules generator. The main contribution of this work is a combinations of semantic rules reasoning and ML reasoning to provide a new ubiquitous context awareness situation framework for healthcare monitoring systems. Those two highly modern and very powerful tools: semantic rules based reasoning and ML based reasoning, should provide complementary and supportive roles in the collection and processing of data, identification of clinical situations and automated decision making for supporting medical activities.

*Paper Organization:* The structure of this paper is as follows: Sect. 2 briefly introduces the related works and background. Section 3 outlines our methodology. Section 4 describes in details our proposed system. Section 5 presents context and situation awareness ontological modelling. Section 6 focuses on the knowledge and reasoning component engine. Section 7 evaluates the proposed system. Concluding remarks and perspectives are presented in Sect. 8.

## Background and Related Works

Adoption of EHR^1^ has increased almost 9 times since 2008 [12]. This huge amount of data circling in clinical information systems has formed new challenges as: semantic interoperability, standardization, automatic medical discovery, knowledge reuse, preventive personalized health services, etc. Aligned with this list of challenges, our work is based on three key concepts: Ontologies, Semantic Rules and ML; presented in the sequel:

**Ontologies Based Semantic Healthcare Modelling:** The conceptual model ontology is encouraging knowledge reuse and simplify problem solving in various fields. Healthcare applications are one of the systems that benefit from using ontologies: drug recommendations discovery [8], clinical support decisions [31], home personalized care to chronic patients [20], healthcare monitoring [33], etc. In Ontologies engineering, integration of ontologies is a useful process that consists on the combination of two or more standard validate ontologies from different disciplines in the aim to create a new multi-disciplinary ontology [23].

**Semantic Rules Healthcare Reasoning:** Semantic web and its technologies are providing efficient solutions in the information and system integration in any distributed information system environments including eHealth systems for which information integration and knowledge discovery are highly recommended [7]. The combination of Semantic Web Rules with Ontology are becoming a mature technology [14]. It use widespread in healthcare and clinical systems. A semantic rules are used in reasoning based approach for dieting and exercising management for diabetics [9]. OWL ontologies and SWRL are combined to integrate reasoning for decision support in alerting system [21].

**ML Techniques for Healthcare:** The high dimensional features and the availability of high quality software made the ML techniques widely used in all fields [4]. It refers to a set of algorithms used to extract useful knowledge or to learn by searching for interesting patterns in a large volumes of previously collected data. The use of ML algorithms in medicine is a hot research topic: disease progression [36, 37], diagnosis prediction [5, 19, 35], and so on. However, those technologies are not mature enough and researchers are still working in the different possibilities and manners to integrate ML algorithms in healthcare systems [29]. One of the combination that appears successful and promising is the combination of ML techniques and ontologies [18, 26, 28].

## Proposed Methodology

Our main goal is the integration of the ML Techniques in a combined ontology semantic modelling and semantic rules based reasoning healthcare framework for chronically ill and elderly patients. Our framework is build up in three keys stage: ontology for semantic modelling and representation, semantic rules for reasoning and machine learning techniques for learning; detailed in the following:

**Semantic Representation and Ontological Modelling:** The aim of this step is to find the best practices in semantic representation for holistic remote health monitoring system that is characterized by a large set of terminologies. Ontology modelling is one of the best choices, and as discussed before, the combination of different standard and valid ontologies is one of the recommended practice to build up an integrated multidisciplinary ontology.

**Semantic Rules Reasoning Based Prediction:** This step consists in the definition of primary knowledge base: a prediction based set of semantic rules. It exists two categories of rules: objectives and subjective. Objective knowledge contains medical rules defined in general medicine textbooks. Subjective knowledge is defined about the patient profile and context such as prior medical history, genetic diseases, personal lifestyle, etc.

**Machine Learning Based Healthcare Reasoning:** The outcome of this step is the best ML algorithm able to give the efficient support to the risk assessment system by providing the best and accurate new medical rules, detailed later in the Algorithm 1.

### Information Life Cycle

**Fig. 1.**
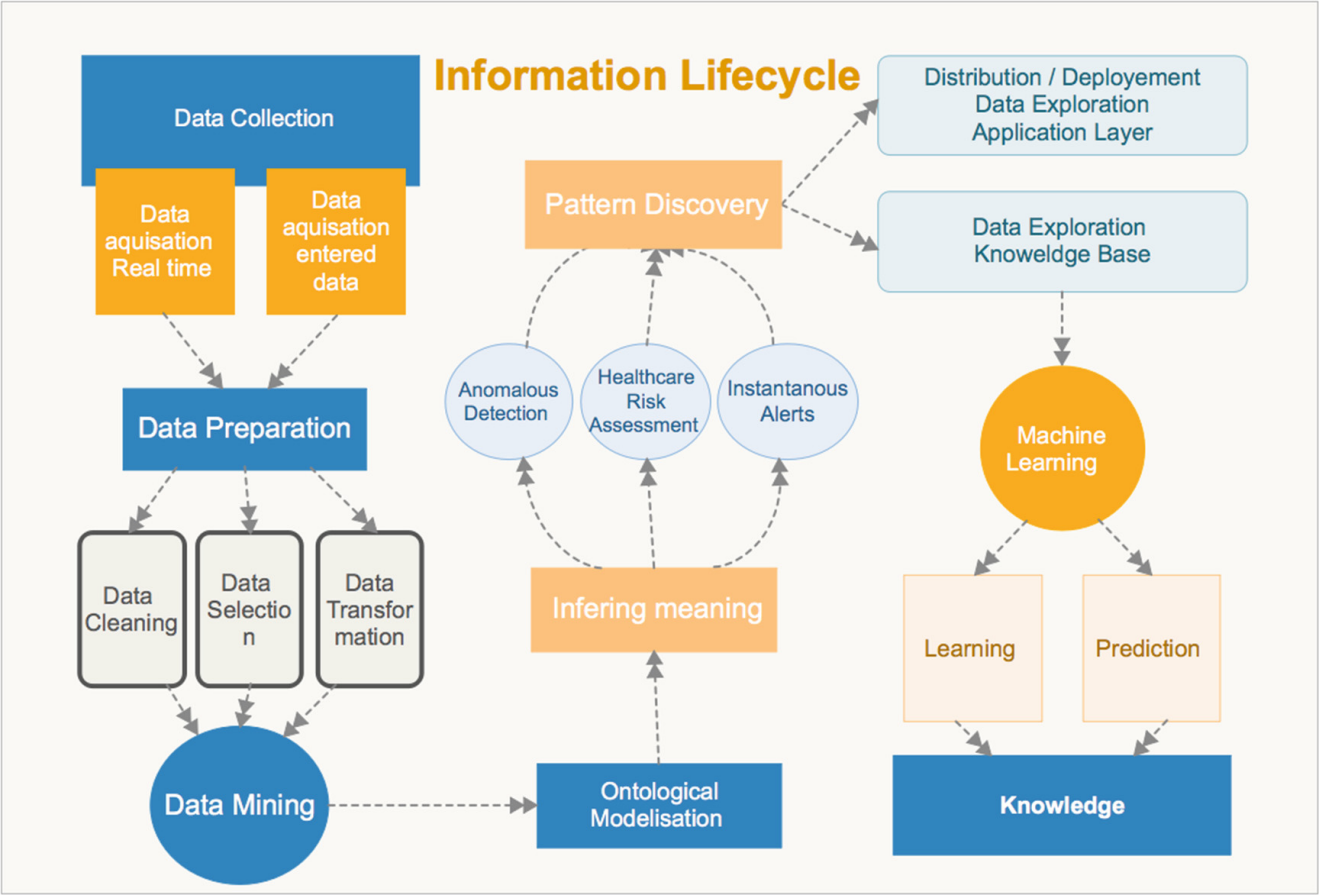
The life cycle of information: data, information, knowledge

In this subsection, we explain the information life cycle in our system: from a data, to an information, then finally a knowledge. The schema of Fig. 1 represents the different steps starting from the collection of data, passing by the different information uses in real-time ubiquitous healthcare monitoring, finally generating of knowledge. We have two main types of data sources: received data from smart devices and entered data by users (medical staff basically). All the data is collected and prepared to be analysed. The first step of the data preparation consists on highlighting the outliers and missing values: any abnormal value could be an alert. Then, in data selection, only contextual and health attributes are selected that are related to the environment or the health situation of a patient. un-selected data will be temporary removed from the data. Different transformations are required, viz, String to Nominal, Unify Date Format. The data mining step is our main contribution because it is not only processed using data mining techniques but also it is based on inferring meaning applied using a set of rules which consists on the subjective knowledge. In a first step, the inferring meaning is used in real-time by the system to determinate the current health situation of the patient, instantaneous alert, healthcare risk assessment and anomalous detection. Each applied rule is registered in the subjective knowledge. This knowledge base is able to grow in terms of number of rules. This growth is assisted by a ML engine. The new learned rules will be dynamically and automatically provided to the objective knowledge. The system should allow the updates of the ML algorithm manually and the updates of the subjective knowledge automatically.

## Detailed System Description

The main functionalities of the system are summarized in the following:

**Electronic Health Record:** is the basic functionality in our system since its the main way of the data collection. It is a systematic recording of contextual and environmental signs adequate to a multimodal, continuous and real-time user interactions.

**Alerts for Instantaneous Rescue:** is the main functionality of our system which consists in providing a help support system for elderly persons with chronic diseases. It awareness users about risks and emergency situations for an early and preventive health care.

**Reasoning for Clinical Decision Support:** is the major contribution of our system providing all necessary techniques that ensure predictive analytic and preventive personalized health services for a better clinical support system.

**Ubiquitous Monitoring and Patient Tracking:** is an optional functionalities provide an instantly monitoring and tracking that may be used for the already mentioned functionalities.

**Fig. 2.**
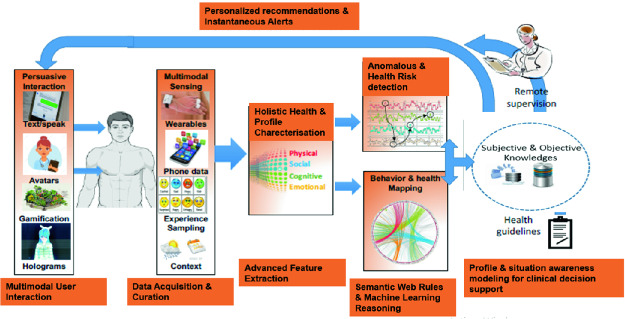
Proposed architecture.

**Patient Reminder:** is an optional but highly recommended functionality. It is hard to imagine a healthcare system that is not providing a patient reminder about medical appointment, daily medical operations, etc. Aligned with the list of proposed functionalities, Fig. 2 is the generic architecture for a combined ML and ontology based situation awareness framework for clinical monitoring and healthcare decision supporting. The main contribution in this architecture is the dynamic updates of its knowledge base in its objective and subjective parts. Our system is a multi modal user interaction application, i.e. different smart devices are available in the patient’s environment or in the patient’s body. The data is collected from different devices like: body sensors worn by the user, ambient sensors surrounding the user or smart devices, as phone, tablet, etc. Body sensors are used to capture the health profile of the patient, viz, vital signs, motion, location, etc. Ambient sensor reflect an image of the patient’s environment, viz, ambient temperature, lightness, existence of a caregiver, etc. Smart devices are basically used to allow the communication between the user and the system and between users, viz sending an alert to user about a patient’s situation, monitoring a patient, etc. The architecture of the proposed system is layered and detailed in the following:

**Active and Assisted Living Sensors Layer:** contains all smart devices including the set of wearable and nearable sensors related directly to the user, his body and his environment. Its role is collecting data for a complete holistic health profile for each patient: health data, ambient data, location, motion, personal information, etc.

**Networking and Communication Layer:** a set of networking device allowing the communication between the different physical elements and the connection of those elements to the internet. **Ontological model based Data Layer:** it contains the set of the collected (current and previous) data and the ontology used in this system.

**Multi-modal Interactions Application Layer:** is the implementation of all the functionalities of the system providing all the services for ubiquitous and continuous medical monitoring and supporting the multi-modal interaction.

**Semantic Rules Based Knowledge Layer:** it is composed by the objective and the subjective knowledge. It is playing a fundamental role in our system since it contains the prediction component, i.e. prevention and detection of emergency cases and alert management.

**ML Based Reasoning Layer:** it is the layer performing the main contribution of our system which is the learning of new predictive and preventive medical or technical rules.

## Context and Situation Awareness Modelling

**Fig. 3.**
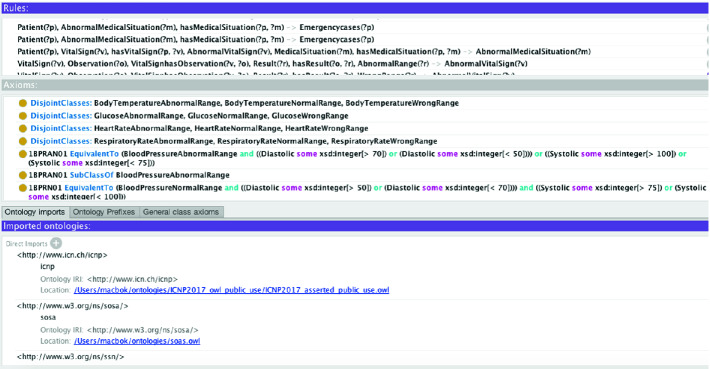
Ontology overview

Our ontology, Fig. 3, is composed from different valid and standard ontologies: ICNP, SSN/SOAS and FOAF. ICNP includes terminologies from the nurses’ statements. And no doubt, nursing science has a significant contribution in healthcare services since nurses’ statements are the early and important step in systematizing and prioritizing healthcare services [10]. In the following, we differentiate predefined classes and properties from ours by prefixing each one of them by the name of its original ontology. As One of the main classes in our application, we consider *icnp* : *Patient* representing the patient, *icnp* : *VitalSign* representing its vital signs and *icnp* : *Result* representing all results of any diagnosis (measurement of vital sign, measurement from blood test, etc). The W3C, SSN is one of the popular ontologies in describing sensors. It describes the sensors capabilities, actuators observation and all the related concepts [3, 27]. SOSA is the lightweight core of SSN that provide general purpose specification model for interaction between sensors. SOSA is an extension of the SSN ontology in semantic web community by providing a flexible framework and easy to use vocabulary [16]. The class *sosa* : *Observation* represents any estimation or calculation of a value of a property of a feature of interest. FOAF declares the person profile in different fields such as health, finance, law, etc. [17]. It has four main categories of information: basic, personal, online accounts and personal documents and images. Those standard and valid ontologies are integrated together: merged, mapped and extended in order to provide the final ontology. In the following, some example of the mentioned operations: **Merging:** in our case this operation has poorly affect our ontology since, it is only linking class with the same name which are not many in the three ontologies. The outcome of this operation a set of equivalent axioms defining equivalence between components having the same name as the entity Person. **Mapping:** we are using FOAF to present the personal profile of users. However, the ontology ICNP has an entity called Individual presenting the health profile of a user as height, weight. So, a merging operation has been performed between the entities FOAF: person and ICNP: individual. **Extending:** The entity Platform from SSN gathers all the entities as sensors, actuators, other platforms hosted in the same platform. We created the property has Platform to link each user to his sensors.

## Knowledge and Reasoning Engine

The knowledge component is defining: general medicine domain and context data using a set of semantic rules SWRL [14] in an abstract manner. So, the semantic rules based knowledge engine will be basically applying such medical knowledge to prevent and detect emergency cases. However, the reasoning techniques will be able to learn dynamically from the previous facts, i.e. previous applied rules, and provide new SWRL rules. The proposed system will allow a self-learning about new relations instances of cause & effect relations between data. Causes are the health situation represented by a set of signs and symptoms. Effects are the medical situation of a patient, viz, emergency case, disease, diagnosis, etc.

### Semantic Rules Knowledge

Reasoner Engine (RE) applies the knowledge into the collected data in the order to determinate new facts about the current patient’s situation in real-time way. RE will be in continuous search of causes which are any changes in the medical situation (signs and symptoms of each patient). Our preliminary set of SWRL is only containing a basic set of medical rules allowing to detect few emergency cases as: less blood pressure, height temperature, etc. To resume any abnormal value for a vital sign, we define the following SWRL^2^ rule (*GM*): *icnp* : *VitalSign*(?*v*) $\wedge$ *sosa* : *Observation*(?*o*) $\wedge$ *VitalSignhasObservation*(?*v*, ?*o*) $\wedge$ *icnp* : *Result*(?*r*) $\wedge$ *sosa* : *hasResult*(?*o*, ?*r*) $\wedge$ *AbnormalRange*(?*r*) $\rightarrow$ *AbnormalVitalSign*(?*v*). The rule is defining abnormal vital signs as the following: for a vital sign ?*v* and its observation ?*o*, if the result ?*r* of the observation ?*o* is a value in an abnormal range, then ?*v* is an abnormal vital sign. *VitalSignhasObservation*(?*v*, ?*o*) and *sosa* : *hasResult*(?*o*, ?*r*) are expressing the relation between a vital sign and its observation and between an observation and its result. The class *AbnormalRange*(?*r*) is representing abnormal ranges.

### Learning and Prediction Reasoning

ML Engine (MLE) learns from previous patient’s detected alarms to produce new reasoning rules in different steps from the Algorithm 1. The MLE is based on the Fast Decision Tree (FDT) Learning Algorithm [34]. Decision Tree (DT) Learning Algorithms are known because of their simplicity, comprehensibility, absence of parameters, and ability to handle mixed-type data. In addition, FDT is a well-known adapted version of DT that scales up well to large data sets with large number of attributes as a healthcare data set.
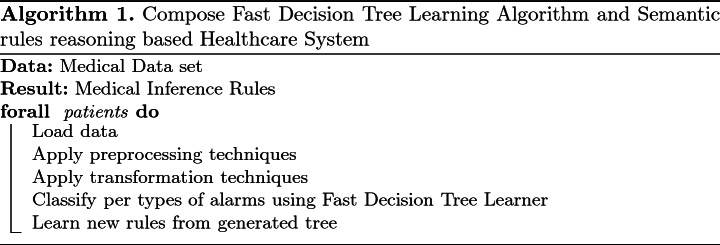


## Performance Evaluation and Results

**Fig. 4.**
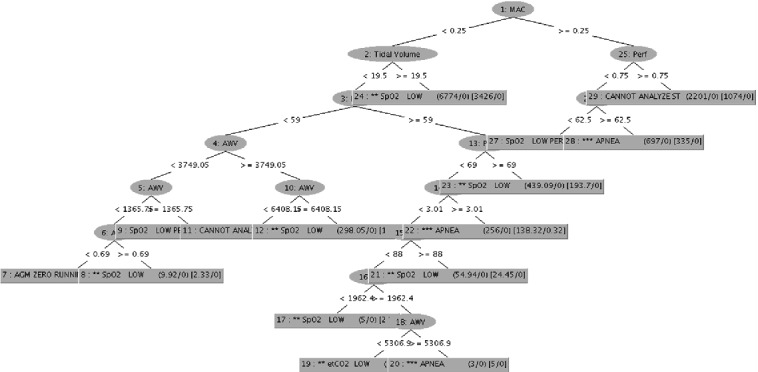
Example of Weka result

In order to prove the feasibility of the proposed approach and to evaluate its performance, we have tested the general process previously proposed in Algorithm 1 to an electronic health recorded data set from [22, 30]. This data is considered as preliminary data set. The different proposed steps have been realised with WEKA [13]. An example of new rule is the detection of a low level of SpO2-Oxygen Saturation- that is used to continuously monitor the oxygenation status of critically ill patients. Actually the SpO2 measurement provides: Pleth waveform (visual indication of patient’s pulse), Oxygen saturation of arterial blood (SpO2) in percent, Pulse rate (derived from Pleth wave), Perfusion indicator (Perf)- numerical value for the pulsatile portion of the measured signal caused by arterial pulsation. Only a medical staff is able to read and to interpret such result, then to detect an emergency case. The result of Algorithm 1 applied to data from one patient allow the definition of the following new rule: Perf < 0.5 and AWV < 3749.05 and AWF < 10.4 and NBP (Sys) < 105 is an emergency case with alarm labelled SpO2 LOW PERF, Fig. 4. Then, the different instances of non normal range of the following signs: Perf, AWV, AWF et NBP will be updated. For example, the following axioms will be defined (or update if it exists previously): **Axiom 1** *PerfAN* *SubClassOf* *PerfAbnoramlRange* and **Axiom 2** *PerfAN* *EquivalentTo* *PerfAbnoramlRange* *and* $some xsd:real[< 0.5]$. Then, the rule *GM* will be applicable to determinate a new alert.

## Conclusions

This paper presents a combined semantic rules reasoning and Fast Decision Tree Learner algorithm for a predictive, preventive and personalized medical framework. The main idea consists in a knowledge and reasoning engine able to apply SWRL medical rules on collected data to generate alerts and able to create new general medicine rules based in previous detected alerts. As a continuation of this work, we are aiming to develop a prototype of the proposed system and test it on a larger samples of data collected from an elderly population with chronic diseases.

## Footnotes

1 Electronic Health Records.

2 We recommend [14] for further information about SWRL notation. The symbol ? is proceeding names of variables and $\wedge$ is the logical And.
